# Geology and lithology of the Tagay-1 section at Olkhon Island (Lake Baikal, Eastern Siberia), and description of Aplodontidae, Mylagaulidae and Sciuridae (Rodentia, Mammalia)

**DOI:** 10.1007/s12549-022-00548-w

**Published:** 2022-11-25

**Authors:** Gudrun Daxner-Höck, Thomas Mörs, Ivan Filinov, Alexander Shchetnikov, Margarita A. Erbajeva

**Affiliations:** 1 Rupertusstr. 16, 5201 Seekirchen, Austria; 2 Natural History Museum Vienna, Burgring 7, 1010 Vienna, Austria; 3 Department of Palaeobiology, Swedish Museum of Natural History, P.O. Box 50007, SE 10405 Stockholm, Sweden; 4 Bolin Centre for Climate Research, Stockholm University, Stockholm, Sweden; 5 Institute of the Earth’s Crust, Siberian Branch, Russian Academy of Sciences, Lermontova str. 128, 664033 Irkutsk, Russia; 6 Geological Institute, Russian Academy of Sciences, Pyzhevsky lane 7, 119017 Moscow, Russia; 7 Laboratory of Geoarchaeology of Baikal Siberia, Irkutsk State University, 5 Armii str. 52, 664025 Irkutsk, Russia; 8 A.P. Vinogradov Institute of Geochemistry, Siberian Branch, Russian Academy of Sciences, Favorskogo str. 1a, 664033 Irkutsk, Russia; 9 Dobretsov Geological Institute, Siberian Branch, Russian Academy of Sciences, Sahianova Str. 6a, Ulan-Ude, 670047 Russia

**Keywords:** Siberia, geology, lithology, geomorphology, Miocene, Rodentia, systematics

## Abstract

Excavations along the Tagay-1 section shed light into the composition of small mammal assemblages of the Tagay site. The present paper focuses on the geology and geomorphology of Olkhon Island, the lithology and fossil evidence along the Tagay-1 section and descriptions of the aplodontid, mylagaulid and sciurid rodents. The described fossils are isolated teeth of four taxa, *Ansomys* sp. (Aplodontidae), *Lamugaulus olkhonensis* Tesakov and Lopatin, 2015 (Mylagaulidae), Sciuridae indet. and *Spermophilinus debruijni* nov. spec. (Sciuridae). The archaic tooth pattern of these rodents suggests an age around the Early/Middle Miocene transition.

## Introduction

**Fig. 1:**
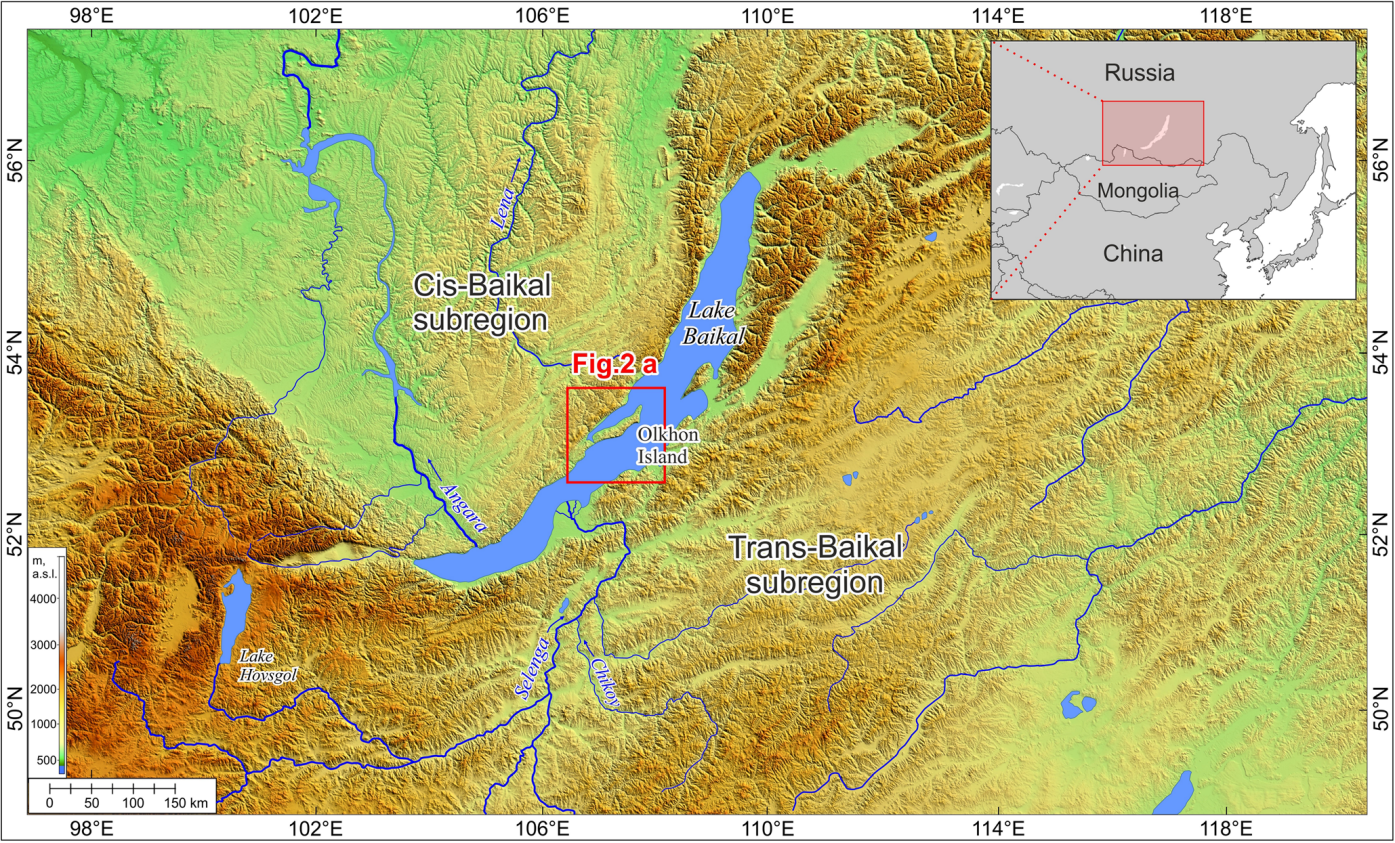
Digital relief of the Baikal region. Data from SRTM v.4 were used in the design of the diagram with a resolution of 90 m. The position of Olkhon Island is indicated by a box (see also Fig. 2a)

Tagay, the most prominent Miocene fossil site on Olkhon Island, is located at Tagay Bay of Lake Baikal, at the north-western part of Olkhon Island. (Fig. 1). The first fossils were discovered by Kitaynik (1958) in the Neogene clays of Tagay, and the lithology and fossils of the Tagay section were published by Logachev et al. (1964) for the first time. Later, additional geological and palaeontological data followed (Mats et al. 1982, 2011; Vislobokova 1990, 1994, 2004; Kossler 2003; Daxner-Höck et al. 2013). In the course of several field campaigns of the Russian Academy of Sciences (principal investigators E.V. Syromyatnikova, A.S.Tesakov and A.M. Klementiev), rich fossil material was collected from the Tagay locality in the 2000s. Numerous articles on the fauna, geology and taphonomy of the Tagay locality were published, others are to be expected (Čerňanský et al. 2020; Klementiev and Sizov 2015; Sizov and Klementiev 2015; Sotnikova et al. 2019; Syromyatnikova 2014, 2015, 2016; Tesakov et al. 2014; Tesakov and Lopatin 2015; Volkova 2020; Zelenkov 2015, 2016, 2018).

Our field campaign of 2014 (principal investigator M.A. Erbajeva) focused on the detailed study of section Tagay-1 across the Tagay Formation (coordinates 53° 9'34.74"N, 107°12'43.12"E). We studied and analysed 17 sediment layers of section Tagay-1 in order to gather information about lithology, sediment structures, fossil content, geochemistry, palaeomagnetics and about dating of the locality and fauna. For better comparison our Tagay-1 section was placed approximately at the same part of the Tagay outcrop, where Logachev et al. (1964: Fig. 12) and Kossler (2003) had studied their Tagay sections. Coordinates of these former sections are not available.

Additionally, we provide in this paper descriptions of the rodent families Aplodontidae, Mylagaulidae and Sciuridae of the Tagay fauna (field campaign 2014 and coll. Kosler). The final biostratigraphic setting of the Tagay fauna will be assessed after completion of the work on all small mammal fossils.

## Geological setting

**Fig. 2:**
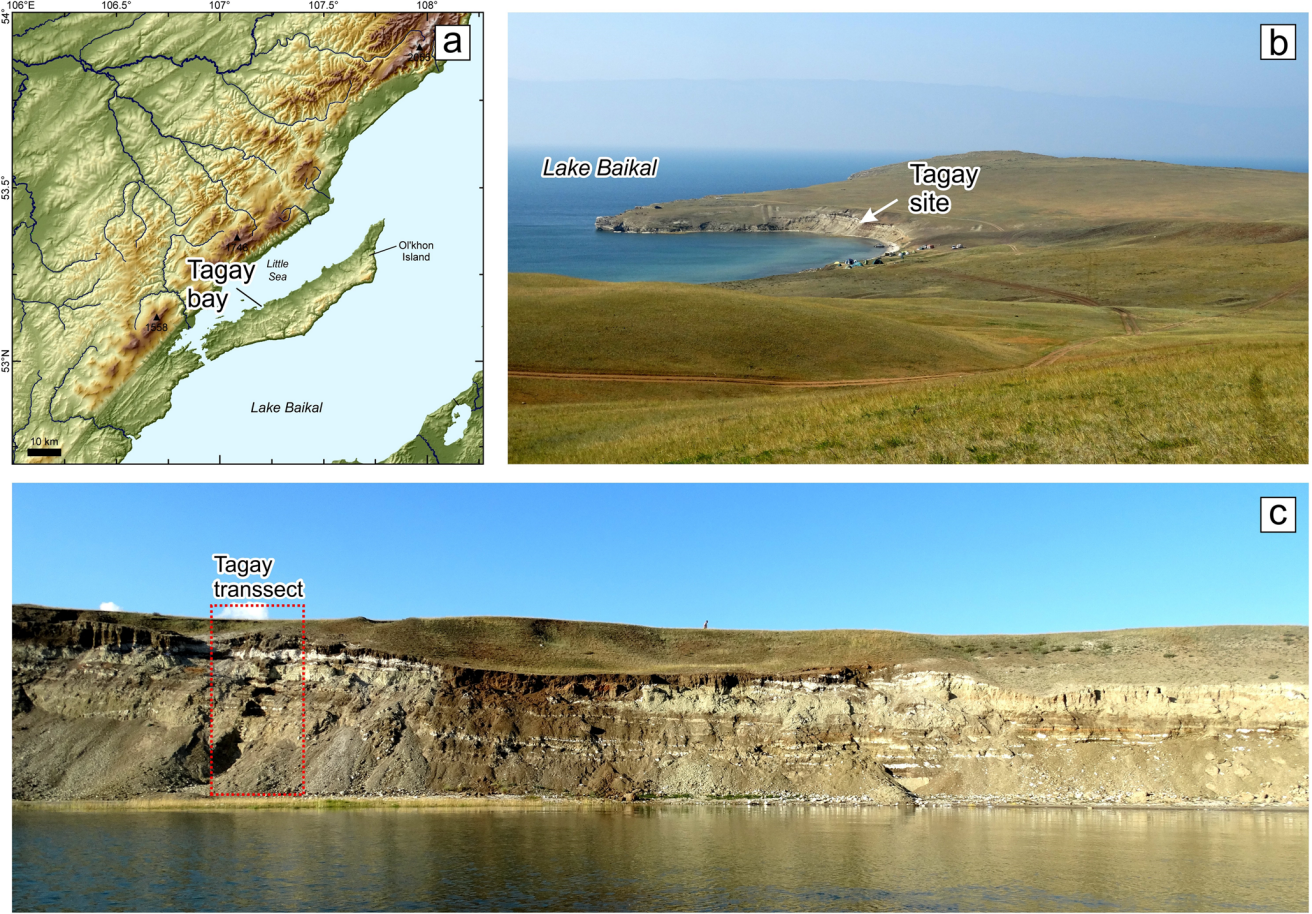
Tagay Bay location on the digital elevation model of the Middle Baikal region (**a**), and typical landscapes of Olkhon Island with a general view from the south-west to the Tagay locality (**b**). The lower photo (**c**) shows the location of the excavation against the general background of the Tagay-1 section, view from the north. Data from SRTM v.4 were used in the design of diagram A. Photos by the authors

**Fig. 3:**
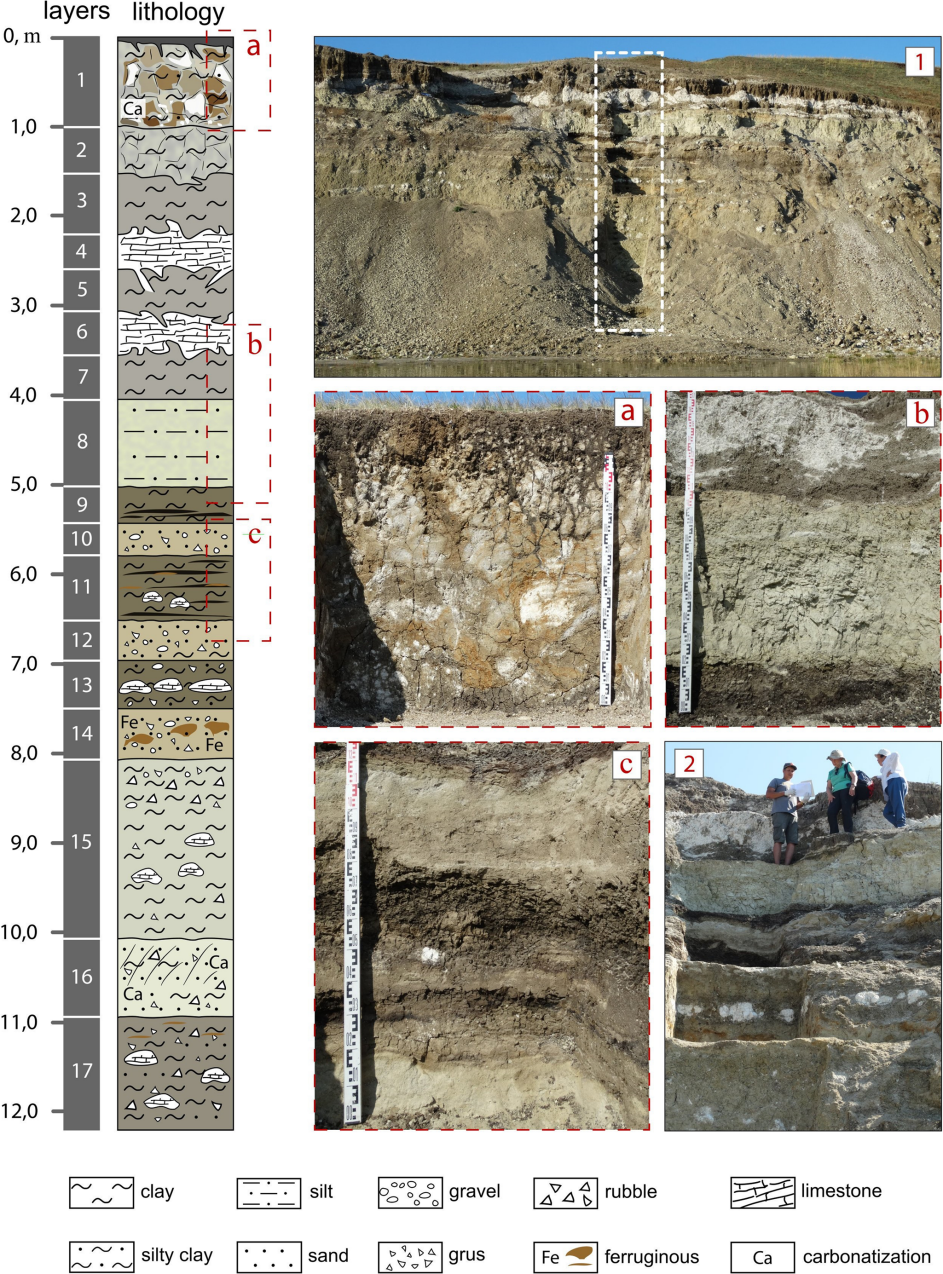
Lithological column of the Tagay-1 section (left) and photos of the “leopard texture” of the weathering crust, formed at the top of the section (**a**), characteristic structures of the middle part of the section (**b** and **c**). The position of the excavation against the general background of the section (**1**) and its upper part, where the main bone-bearing horizons are concentrated (**2**). Coordinates 53° 9'34.74"N, 107°12'43.12"E. Photos by the authors

Olkhon Island is the biggest island of Lake Baikal (Fig. 1) and part of the Baikal National Park, which is included in the list of the UNESCO World Heritage Sites. It stands out in the Baikal region for many of its notable features, including its geological and geomorphological conditions. One of the main features is the unique relief of the island. The relics of the ancient, Cretaceous-Paleogene peneplain (Fig. 2) with fragments of small Neogene basins, have been preserved here practically in their original shape. Before the onset of Miocene sedimentation, the relief of Olkhon Island had an almost modern appearance. It is demonstrated by the island bedrock massifs protruding from under the Neogene deposits with undeformed Miocene sediments leaning against their basin slopes (Logachev 1974). The relatively thick profile of the weathered crust (eluvium) was built along the surface of the Tertiary depressions (Fig. 3, layer 1).

The bedrocks of the section are represented by Paleozoic metamorphic complexes, mainly biotite gneisses and migmatites with deep pockets of the weathering crust (Mats et al. 2001). The depressions of the crystalline basement are covered by a thin layer of Neogene sediments in the form of variegated, in the lower part of the section, essentially lithified, sandy clays, loams and silts with horizons of pelitomorphic carbonate formations (limestones) and numerous included fossils.

Neotectonic processes are weak on Olkon Island compared with other parts of the Baikal region. During the Pleistocene, the tectonic block of the island, bounded by listric faults, began to sink with an inclined surface on the western flank (Mats 1993; Ufimtsev 1993), where part of the island subsided into Lake Baikal. This sinking process is accompanied by the formation of tectonic shores of the island.

As a consequence, an ordered alternation of wide open, rectangular shaped bays is observed here, jutting into the land usually for 1.0–1.5 km and of the same shape and usually with wider peninsulas and promontories. Simultaneously, depressions with small lagoons embedded, are often located in the necks of the peninsulas. It also shows evidence of a young lacustrine ingression.

Thus, the coastal zone of Olkhon Island in these areas is under the influence of abrasion-tectonic destruction: blocks of the crystalline basement in the form of bays gently submerge under the water of the lake, simultaneously being exposed to coastal erosion, and forming cliffs by abrasion. In one of these cliffs, the sedimentary sequence of the Tagay section is exposed (Fig. 2b).

In addition, the distortions of the topographic surface of Olkhon Island also cause the activation of landslide processes, which are widespread in the amphitheatre of the coastal zone of Tagay Bay. However, the Tagay location itself was not affected by landslide disturbances.

## Lithology of the Tagay-1 section

In 2014, the Tagay-1 section was studied in order to find fossil concentrations along the section, and to compare the fossil content with data published before. The layers of the section have an irregular course, by inclining towards the southwest, sometimes pinching out (Fig. 2). Consequently, we decided to locate our new section close to the one published by Logachev (1964). It allowed reliable correlation of the stratigraphic sequence, comparison of the lithologies of sediment layers and the fossil content of both the published and the new section.

The secton was documented and sampled for various types of analytical studies. Below is a short layer-by-layer description of the Tagay-1 section (from top to bottom, Fig. 3):

### Description of section Tagay-1

**Layer 1:** Variegated (red, white, grey, brown colours) sandy clays with chaotic spots and lenses of silt (leopard texture), cracks filled by modern soils. The lower boundary is not clear.

Thickness 0.7-1.0 m.

**Layer 2:** Light brown and dark grey clay and silt with spots of carbonate, with admixture of sand. Lower boundary irregular with sediment pockets cutting into layer 3.

Thickness 0.4-0.6 m.

**Layer 3:** Dark green clay, with numerous inclusions of white carbonate concretions. Lower boundary sharp, slightly undulating. *Fossil remains*.

Thickness 0.5-0.7 m.

**Layer 4:** Large limestone concretions in an argillaceous pelite with dark (almost black) clay spots. Thickness of the layer variable. Irregular lower boundary, with depressions into the underlying sediments.

Thickness 0.2-0.8 m.

**Layer 5:** Yellowish-green, slightly sandy clay with many cracks. Lower boundary irregular with deep intrusions into the underlying layer. *Many fossil remains.*

Thickness 0.5 m.

**Layer 6:** White limestone pelite, sandy, in part with carbonate concretions. Lower boundary irregular with deep depressions into the underlying layers. *Fossil remains.*

**Layer 7:** Dark green sandy clay, with many chaotic white carbonate spots. Thickness variable. Lower boundary sharp, slightly undulating. *Fossil remains*.

Thickness 0.2-05 m.

**Layer 8:** Light green homogeneous silt, in upper part brownish and slightly waved. Lower boundary sharp, slightly undulating, occasionally with small lenticular, spotted injections into the underlying layer.

Thickness 1.0 m.

**Layer 9:** Brown and dark brown clay, bedding clear due to colour changes. Upper part and lower part are slightly lighter in colour. Lower boundary sharp, slightly undulating. *Many fossil remains*.

Thickness 0.4 m.

**Layer 10:** Yellowish-green inequigranular sand and gravel, unsorted, without bedding. Lower boundary sharp. *Some fossil remains*.

Thickness 0.4 m.

**Layer 11:** Brown clay, bedded, sandy layer replaced by clay beds, with horizon of carbonate concretions and lenses of orange-ochre sandy clay. Lower boundary sharp, slightly undulating. *Some fossil remains.*

Thickness 0.8 m.

**Layer 12**: Yellow-grey inequigranular sand with gravel. Lenses of dark-green clay. In the bottom layer poorly sorted clay and sand. Lower boundary sharp, slightly undulating.

Thickness 0.4 m.

**Layer 13:** Dark green clay with horizon of white limestone concretions. In the upper and lower parts of the layer there are also inequigranular sand and gravel. Lower boundary sharp, slightly undulating. *Rare Fossils*.

Thickness 0.6 m.

**Layer 14:** Yellowish-grey inequigranular sand and gravel. Not sorted, not bedded. Ferruginous lenses. Lower boundary sharp, slightly undulating.

Thickness 0.5 m.

**Layer 15:** Light-green clay, compact, with debris, gravel. In upper part highest concentration of gravel and sand. In middle part there are spots of white limestone. Lower boundary irregular.

Thickness 2.1 m.

**Layer 16:** Light green clayey sand with spots of limestone, slightly ochrous, with debris gravel. Lower boundary irregular.

Thickness 0.8 m.

**Layer 17:** Dark green silty clay, very compact, sandy, with gravel, concretions of white limestone.

Thickness 1.3 m.

## Material and methods

**Fig. 4:**
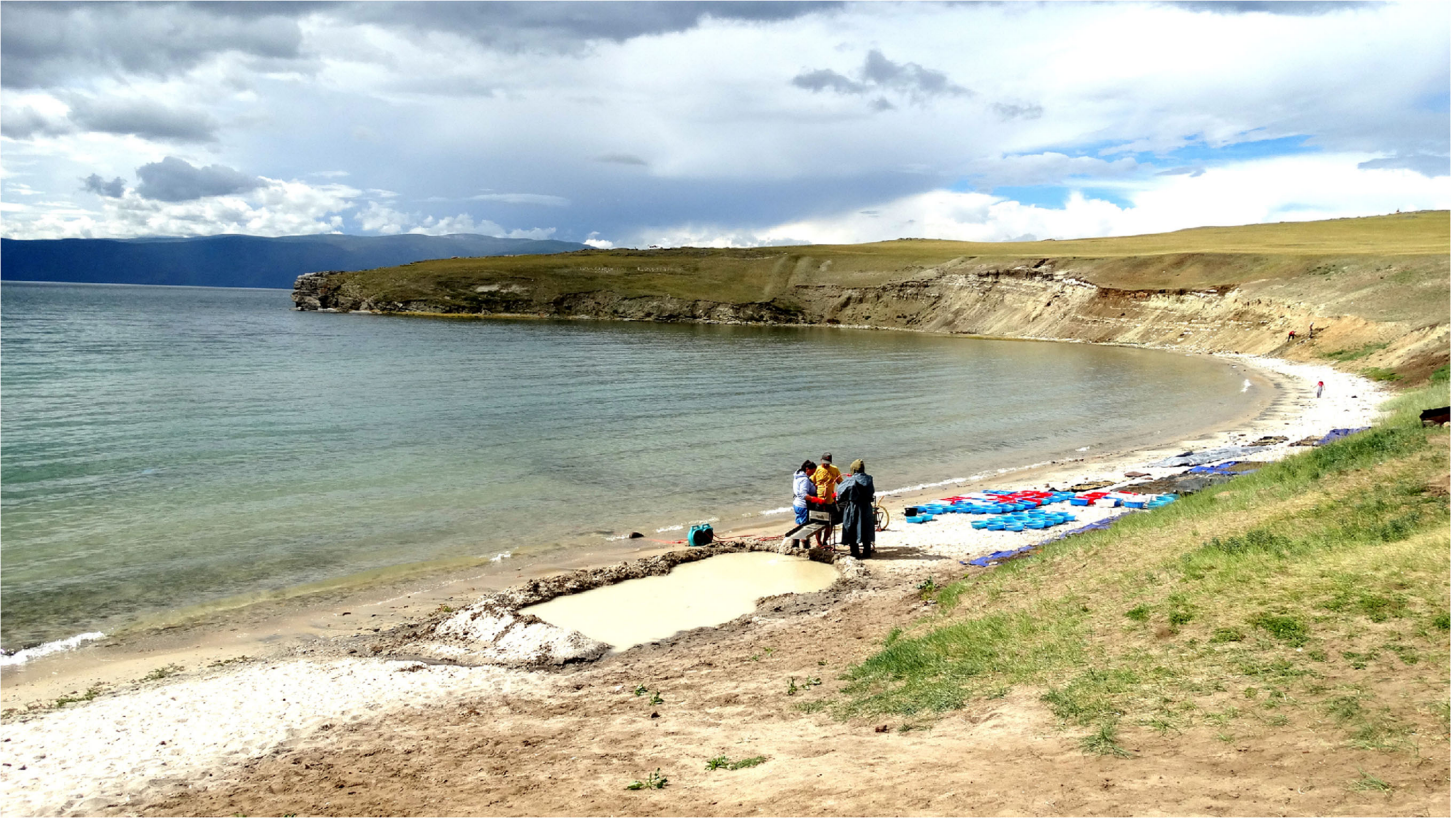
The wet screening process at Tagay site. Photo by the authors.

In the course of excavations of the Tagay Formation (Mats et al. 2011) along section Tagay-1 we collected test samples of approximately 100 kg sediment of each defined sediment layer (Fig. 3) in order to find scattered fossils or fossil concentrations. After drying, these samples were wet-screened, using a washing table with sieve sets of 0.5, 2.5 and 5.0 mm mesh sizes (Fig. 4). Finally, the fossils were picked out from the residuals by using head lenses for the coarse fraction, and binoculars for the fine fraction. From beds with positive fossil record we collected additional samples. The total amount of investigated sediment was ~ 2000 kg.

It turned out, that layers 3, 5, 6, 7, 8, 9, 10 and 11 yielded fossil remains of snails and/or ectothermic vertebrates, and small mammal fossils. Diagnostic teeth, mandibles and maxillary fragments of small mammals were collected from these beds, except for layer 3. They represent lagomorphs, eulipotyphlans and rodents.

SEM images were made by Daniela Gruber [Core Facility Cell Imaging and Ultrastructure Research, University of Vienna - member of the Vienna Life-Science Instruments (VLSI)] by using the Scanning Electron Microscope (SEM JEOL IT 300 LV LaB6) and Sputter coater (JEOL JFC-2300HR).

**Fig. 5:**
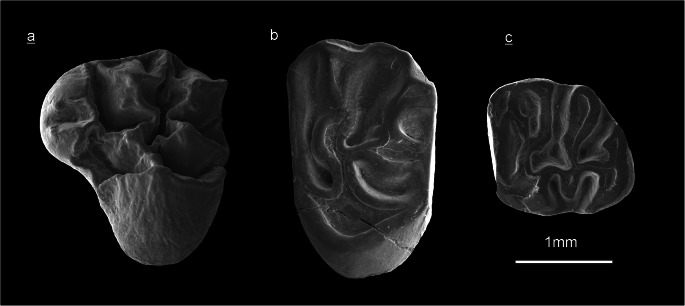
*Ansomys* sp. from the Tagay-1 section (layers 9 and 7; Olkhon Island, Baikal Region, Siberia). All teeth are in occlusal view. **a** Right P4 (ZIN 106466), layer 9. **b** Left M1/2 (ZIN 106467), layer 7. **c** Right m3 (ZIN 106468), layer 7. (underlined = right, not underlined = left).

To facilitate comparisons all right-side teeth are figured as mirror images (reversed = as if they were from the left side), and their figure letters are underlined (e.g. Fig. 5a shows the P4 from the right side). All measurements are given in mm.

The fossils studied here were collected during two field campaigns at the Tagay locality: One part was collected by A. Kossler in the 1990s. It comprises gastropods and fish-teeth and few remains of frogs, turtles, lizards and mammals (Kossler 2003; Daxner-Höck et al. 2013). These fossils are housed in three collections: The small mammal remains are integrated in the collection of the Natural History Museum, Geological-Paleontological Department, Vienna (NHMW), whereas the ectothermic vertebrates are deposited in the Bavarian State Collection for Palaeontology and Geology in Munich (BSPG), and the gastropods in the collection of the Freie Universität Berlin, Section of Palaeontology. The second part was collected in the course of the field campaign in summer 2014 by M. Erbajeva and her team. After publication the fossils of field campaign 2014 will be stored in the collection of the Zoological Institute of Russian Academy of Sciences (ZIN), St. Petersburg.

For classification above genus level we follow McKenna and Bell (1998). Description of Aplodontidae teeth follows the terminology used by Qiu and Li (2016). The description of the Mylagaulidae tooth follows the terminology used by Rensberger (1979), differing terms used by Tesakov and Lopatin (2015) are marked by an*.

Abbreviations

NHMW: collection of the Natural History Museum Vienna (Austria)
ZIN: collection of the Zoological Institute of Russian Academy of Sciences, St. Petersburg.
P – M: premolars and molars of the upper dentition
p – m: premolars and molars of the lower dentition
I – i: upper and lower incisors
n: number of specimens
MN: European Neogene Mammal Zone (Steininger 1999)
LMS/A: Chinese Land Mammal Stage/Age (Qiu et al. 2013)
Ma: million years

**Systematic Palaeontology**

Class Mammalia Linnaeus, 1758

Order Rodentia Bowdich, 1821

Superfamily Aplodontoidea Brandt, 1855

Family Aplodontidae Brandt, 1855

Subfamily Ansomyinae Qiu, 1987

Genus *Ansomys* Qiu, 1987

*Ansomys* sp.

(Fig. 5, Tab. 1)

**Table 1:** Measurements of *Ansomys* sp. teeth of the Tagay-1 section (layers 7 and 9; Olkhon Island, Baikal region, Siberia).

*Ansomys* sp.					
object	coll. number	length	width	Tagay-1	Fig.
P4 right	ZIN 106466	2.02	2.09	layer 9	5a
M1/2 left	ZIN 106467	1.55	2.52	layer 7	5b
m3 right	ZIN 106468	1.50	1.45	layer 7	5c

**Locality, Stratigraphy:** The Tagay-1 section (layers 9 and 7; sample 2014) from Olkhon Island, Baikal Region, Siberia; Tagay Formation (Mats et al. 2011); Early/Middle Miocene transition.

**Material:** Three teeth (ZIN 106466 – ZIN 106468) from layers 9 and 7 of the Tagay-1 section, collected by Erbajeva and colleagues in 2014.

**Description:** The material comprises three teeth, largely following the morphological characteristics of the genus *Ansomys.* All teeth are brachyo-lophodont. Unworn teeth show crested loph(id)s and cones(conids). Upper teeth have: a dominating protocone, no hypocone, a double crested mesostyle forming a labial peak, which is part of the sharply crested ectoloph. Protoconule and metaconule are single. Lower teeth have a clearly lophodont character. The main conids are lophid and compressed antero-posteriorly. Mesoconid and hypoconulid are large and triangular. Mesostylid and metaconid are connected by the metastylid crest. Anterolophid, mesostylid crest + metalophid II, hypolophid and posterolophid are transversely directed. Except for metalophid I, no accessory crests are visible in the valleys.

**P4 right** (Fig. 5a) consists of the unworn crown; no roots are preserved. The occlusal surface is triangular, and widest in its labial part. P4 has seven peaked cones, each has three sharp crests and is triangular in cross section. All these cones are of almost equal size and elevation except for the prominent protocone, which is highest and extended in antero-posterior direction. Paracone and metacone have convex labial and lingual walls and are concave at the posterior and anterior sides, respectively. Protoconule and metaconule are placed at the middle part of protoloph and metaloph; they have flat labial and convex lingual walls and are also concave at the posterior and anterior sides, respectively. The anterior double-cone is anteriorly split by a deep notch separating the anterocone from the anterocone style. The anterior walls of this double cone are convex, all others flat. The protocone is elongated in antero-posterior direction; in the anterior corner it fuses at 90° with the short anteroloph, posteriorly it ends freely at the posterior edge. No hypocone exists. Protoloph and metaloph, appearing as W-shaped sharp crests, fuse lingually to one loph before reaching the protocone. No posteroloph exists. The double crested (= bifid) parastyle and mesostyle are continuous with the labial crests of paracone and metacone, and appear as sharp ectoloph. The sharp posterior crests of metacone and metaconule extend to the posterior margin. The anterior crest of protoconule (protolophule) contacts the double anterocone immediately opposite to the notch. All valleys between crested lophs and cones are deep and narrow.

**M1/2 left** (Fig. 5b) is strongly worn, showing a flat occlusal surface. The occlusal outline is rectangular, wider than long, showing flat wear facettes of the paracone, metacone and the dominant protocone. Differing from P4, the M1/2 has no W-shaped but an almost transverse protoloph, showing facettes of paracone and a weak protoconule. The metaloph is obliquely directed, it ranges from the metacone at the labial margin toward the tooth centre and fuses with the protoloph, finally, the joint loph attaches with the protocone. The metaconule appears as a large wear facette ranging from the metaloph to the posteroloph. The large wear facette of the antero-posteriorly elongated protocone connects the weak posteroloph and the transverse anteroloph (= anterior cingulum). The handle-shaped ectoloph extends from the paracone to the metacone, incorporating the weak mesostyle and the mesostyle crest. Three roots are preserved, one lingual, and two very small labial ones.

**m3 right** (Fig. 5c) is of medium wear stage. It is longer than wide, lophodont, has two transversely elongated main conids (metaconid and entoconid), the large antero-posteriorly elongated protoconid and four transverse lophids (anterolophid, mesostylid crest fused with metalophid II, hypolophid, short posterolophid). A short additional lophid (metalophid I) connects the metaconid with the metalopid II. The position of the entoconid is anterior to the hypoconid. The longitudinally directed ectolophid shows a pronounced mesoconid, which is elongated in labial direction by the mesoconid crest (= ectomesolophid). The labial crest of the hypoconid turns forward but does not contact the mesoconid crest. The hypoconulid has no connection with the hypolophid. The symmetric labial sinusid is bound by the hypoconid, the ectolophid and the protoconid, and is subdivided by the mesoconid crest into an anterior and a posterior part. The shallow trigonid basin is divided by the metalophid I into a labial and a lingual part. The transversely oriented talonid basin is lingually open. The third lingual basin, the posterolingual fossettid, is also open toward the postero-lingual edge of the tooth. There are no accessory crests in the valleys. The m3 is smallest of the three available teeth. Its identification as last lower molar is indicated by the short posterolophid, the flattened postero-lingual corner, and three roots (a large posterior and two small anterior roots).

***Ansomys*** **species from North America, Europe and Asia and their stratigraphic ranges:** Oligocene and Miocene species of the cosmopolitan genus *Ansomys,* distributed over the Old and New World, were discussed by Qiu and Li (2016: 535-538). Some of them are: *A. descendens* (Dehm, 1950) (= former ?*Plesispermophilus descendens* Dehm, 1950) (Wintershof-West, Germany; Burdigalian, Early Miocene, MN3), *A. shantungensis* (Rensberger and Li, 1986) = former *Prosciurus*? *shantungensis* Rensberger and Li, 1986 (Shantung, China; late Oligocene), *A. orientalis* Qiu, 1987 (Sihong locality, China; Shanwangian LMS/A, Early Miocene), *A. shanwangensis* Qiu and Sun, 1988 (Shanwang, China; Shanwangian LMS/A, Early Miocene, around 18 Ma), *A. nexodens* (Korth, 1992) (= former *Pseudallomys nexodens* Korth, 1992) (Montana, USA; Orellan, early Oligocene), “*A. crucifer* Lopatin, 1997” [excluded from the genus *Ansomys* by cladistic analyses of Hopkins (2004: 731-733)] and “*A. daxnerae* Lopatin, 2000” [= synonym of *Proansomys daxnerae* (Lopatin, 2000) according to Maridet et al. (2017: 42)]. The two latter taxa are from the late Oligocene of Altynshokysu, Kazakhstan [= Early Miocene according to Lopatin (2000)], *A. hepburnensis* Hopkins, 2004 (Montana, USA; Barstovian (Ba2), Middle Miocene), *A. borealis* Qiu and Li, 2016 (Nei Mongol, China; Early to Middle/? Late Miocene), *A. robustus* Qiu and Li, 2016 (Nei Mongol, China; Early Miocene) and *A. lophodens* Qiu and Li, 2016 (Nei Mongol, China; Middle to Late Miocene).

Moreover, two unspecified *Ansomys* occurrences were described by Maridet et al. (2017) as *Ansomys* sp.1 (Huch Teeg, Mongolia; Early Miocene) and *Ansomys* sp. 2 (Ulan Tolgoi, Mongolia; Early or Middle Miocene).

**Discussion:** *Ansomys*, a brachyodont genus of the Aplodontidae, is generally rare in mammal assemblages of Eurasia. With a total stratigraphic range from the Oligocene to the Late Miocene in Eurasia it is best known from Early and Middle Miocene assemblages of Nei Mongol in China. Its morphological affinities and differences and a possible descent from *Proansomys* Bi et al., 2013 was discussed by Qiu and Li (2016: 536). The genus *Proansomys* is known by three species: *P. dureensis* Bi et al., 2013 (late Oligocene, Junggar Basin, China), *P. badamae* Maridet et al., 2017 (late Oligocene, Valley of Lakes, Mongolia) and *P. daxnerae* (Lopatin, 2000) (late Oligocene, Aral Region, Kazakhstan), all differing from *Ansomys* by a more archaic tooth morphology [Bi et al. (2013: p. 5-6, fig. 5), Qiu and Li (2016: 536) and Maridet et al. (2017: 42)].

*Ansomys* sp. of the Tagay-1 section is similar to the type species *A. orientalis* from the Early Miocene of China but seems more advanced. From the Middle to Late Miocene species of China it essentially differs by lower tooth crowns and less complex molar pattern (no accessory crests of lower molars). Because of the poor fossil record, the Siberian specimens are described in open nomenclature.

Family Mylagaulidae Cope, 1881

Subfamily Promylagaulinae Rensberger, 1980

Genus *Lamugaulus* Tesakov and Lopatin, 2015

**Fig. 6:**
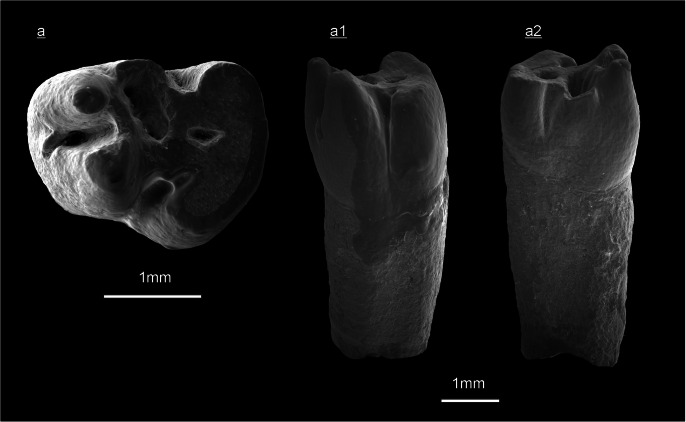
*Lamugaulus olkhonensis* Tesakov and Lopatin, 2015 of the Tagay-1 section (layer 9; Olkhon Island, Baikal region, Siberia). **a** occlusal view, **a1** labial view, **a2** lingual view of right p4 (ZIN 106456)**.** (underlined = right).

*Lamugaulus olkhonensis* Tesakov and Lopatin, 2015 (Fig. 6)

**Locality, Stratigraphy**: The Tagay-1 section (layer 9) from Olkhon Island, Baikal Region, Siberia; Tagay Formation; Early/Middle Miocene transition.

**Material and measurements:** One right p4 (ZIN 106465) of layer 9 from Tagay-1 section, collected by Erbajeva and colleagues 2014. Length = 2.38 mm, width = 2.02 mm, lingual crown hight = 2.38 mm, labial crown hight = 2.53 mm.

**Description:** The crown of the lower p4 (Figs 6a, a1, a2) is about as high as long, and the width/length ratio is 0.85. The tooth has one massive root with almost oval cross-section. The slightly worn p4 shows four main conids, the metaconid and protoconid in anterior position, the hypoconid and entoconid in posterior position. The anterior conids are cusped and higher than the posterior ones. The longitudinally directed ectolophid, showing a mesoconid, connects the protoconid and hypoconid, respectively. The slightly worn posterior conids are connected by the posterolophid and the hypolophid, forming a talonid plateau of lower elevation, in which the shallow posterior fossettid (= metafossettid *) is embedded. A second fossettid, the lingual fossettid (= mesofossettid *) is a deep funnel, surrounded by the hypolophid, the lingual metastylid crest and the metalophid II (= protolophid *). The latter connects the anterior pair of conids. The anterior inflection (= anteroflexid *) between metaconid and protoconid is not closed anteriorly because of the low wear stage. Consequently, the p4 from Tagay has no anterior fossetid in the present wear stage. The labial inflection (= hypoflexid *) narrows towards the base of the crown and extends close to the lower boundary of the crown (Fig. 6a1). The lingual inflection (= mesoflexid *) also narrows and reaches almost half of the crown height (Fig. 6a2). The anterior inflection (= anteroflexid *) also reaches half of the crown hight, and shows a small conulid at the base of the metaconid (Fig. 6a).

**Remarks/discussion**: The first Asian Mylagaulidae record was *Tschalimys ckhikvadzei* from the Middle Miocene of the Zaisan Depression in Kazakhstan (Shevyreva 1971). Subsequently, *Sinomylagaulus halamagaiensi* was described from the Middle Miocene of the Junggar Basin in China (Wu 1988), and later recognised as synonym of *T. ckhikvadzei* (Wu et al. 2013). The most recent findings of *Tschalimys* cf. *T. ckhikvadzei* were recorded from the Early and Late Miocene of Nei Mongol in China (Qiu and Li 2016), and two new species of a new genus, *Irtyshogaulus minor* and *Irtyshogaulus major*, have been described from the Early Miocene of the Junggar Basin (Lu et al. 2016).

From the Tagay locality, the first Mylagaulidae fossils were collected by Tesakov and his team in summer 2012 and described as *Lamugaulus olkhonensis* Tesakov and Lopatin, 2015. The presently studied right p4 was excavated during the field campaign 2014 of Erbajeva and colleagues from the Tagay-1 section (layer 9) of the same Tagay locality. It is closely comparable with the tooth pattern and size of the type material (Tesakov and Lopatin 2015: 24, fig. 1c), and is presently described as *L. olkhonensis*. Hitherto, this species is only known from the Tagay locality, and represents one of the rare Mylagaulidae finds in Asia.

Following Tesakov and Lopatin (2015: 23), the relatively small, mesodont prismatic, single-rooted cheek teeth with five fossettes on upper, and three fossettids on lower cheek teeth indicate *Lamugaulus* as an Asian genus of the subfamily Promylagaulinae, one of two subfamilies of the Mylagaulidae family. Promylagaulinae are known to range in North America from the early Arikareean (Ar1, late Oligocene) to the Barstovian (Ba1, Middle Miocene) (McKenna and Bell 1998; Flynn and Jacobs 2008). They likely dispersed to Asia via the Bering bridge in the course of the Early Miocene. Tesakov and Lopatin (2015) suggest an Early Miocene age of *L*. *olkhonensis* from the Baikal region.

Family Sciuridae Fischer, 1817

Subfamily Sciurinae Fischer, 1817

Genus *Spermophilinus* de Bruijn and Mein, 1968

*Spermophilinus debruijni* nov. spec.

(Fig. 7, Tab. 2)

**Fig. 7:**
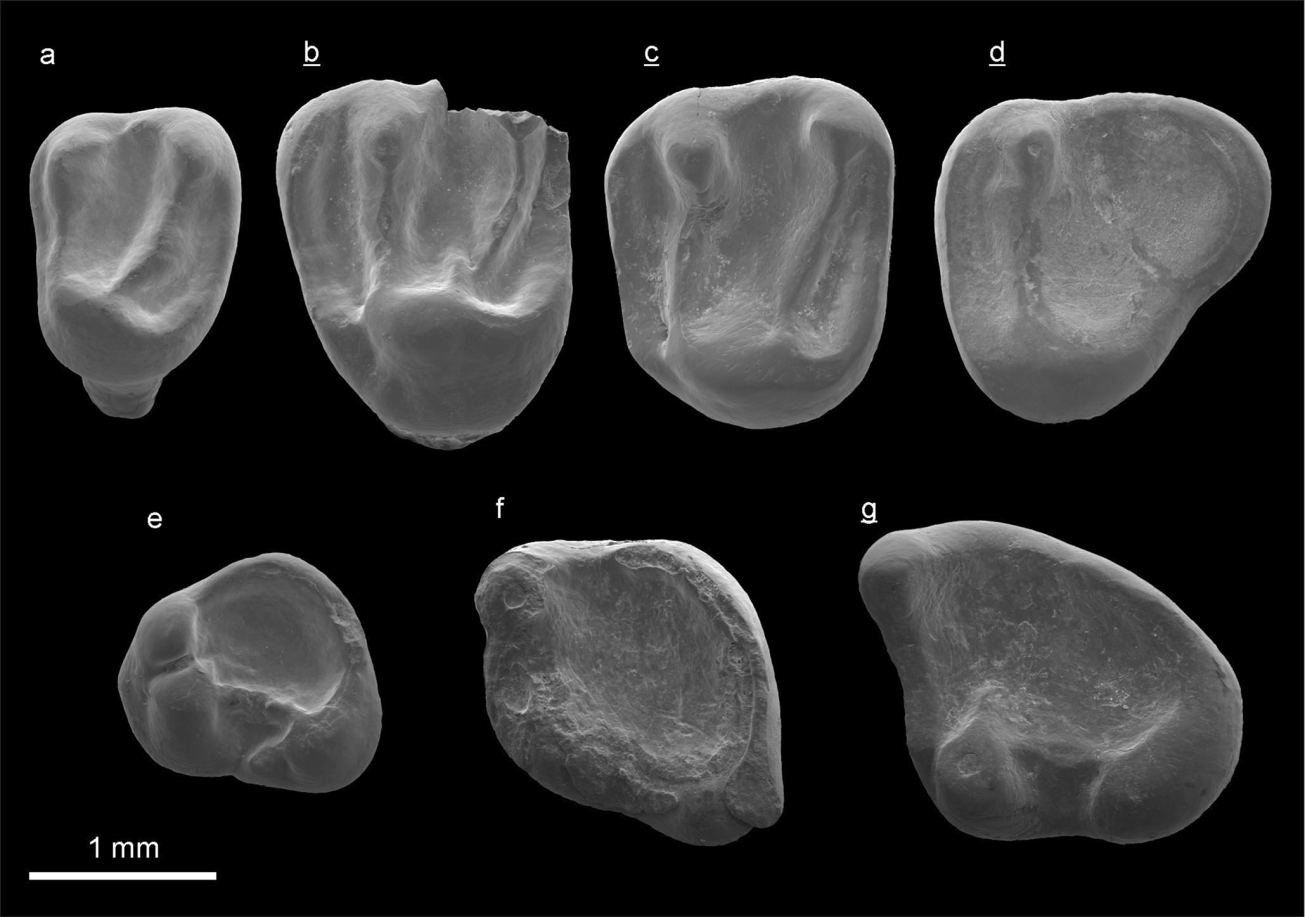
*Spermophilinus debruijni* nov. spec*.* of the Tagy-1section (layers 9 and 7) and from test sample Kossler (Ko) (Olkhon Island, Baikal Region, Siberia). All teeth are in occlusal view. *H* holotype. **a** Left P4 (NHMW2009/0069/0001), Ko. **b** Right M1/2 (ZIN 106456), layer 9. **c** Right M1/2, **H** (ZIN 106455), layer 9. **d** Right M3 (ZIN 106458), layer 7. **e** Left p4 (ZIN 106460), layer 7. **f** Left m1 (ZIN 106461), layer 7. **g** Right m2/3 (ZIN 106462), layer 7. (underlined = right, not underlined = left).

**Table 2:** Measurements of *Spermophilinus debruijni* nov. spec. teeth of the Tagay-1 section (layers 9 and 7) and test sample Kossler (Ko) (Olkhon Island, Baikal region, Siberia). H= holotype, P= paratypes.

*S. debruijni* nov. spec.					
object	coll. number	length	width	Tagay-1	Fig.
P4 left	NHMW2009/0069/0001	1.14	1.45	Ko	7a
M1/2 right **H**	ZIN 106455	1.55	1.86	layer 9	7c
M1/2 right (**P**)	ZIN 106456	1.60	1.93	layer 9	7b
M1/2 right (**P**)	ZIN 106457	1.40	1.90	layer 7	
M3 right (**P**)	ZIN 106458	1.83	1.76	layer 7	7d
p4 left (**P**)	ZIN 106459	1.31	1.14	layer 9	
p4 left (**P**)	ZIN 106460	1.36	1.24	layer 7	7e
m1 left (**P**)	ZIN 106461	1.62	1.62	layer 7	7f
m2/3 right (**P**)	ZIN 106462	1.81	1.76	layer 7	7g
m2/3 left (**P**)	ZIN 106463	1.85	1.78	layer 7	

2013 Sciurinae gen. et spec. indet*.* – Daxner-Höck, Böhme and Kossler: 511, Plate 22.1, fig. 5.

**Etymology:** The species is named to honour of Hans de Bruijn, one of the greatest mammal Palaeontologists.

**Holotype:** (Tab. 2; Fig. 7c). Right M1/2 (ZIN 106455).

**Paratypes:** (Fig. 7b, d, e, f, g Tab. 2). 9 cheek teeth (ZIN 106456 – ZIN 106463).

All type specimens (ZIN 106455 – 106463) were collected by Erbajeva and colleagues from layers 9 and 7 of the Tagay-1 section in 2014.

**Additional material** (Tab. 2; Fig. 7a): One left P4 (NHMW 2009/0069/0001) from a test sample collected by Kossler in the 1990s from the Tagay locality (Kossler 2003; Daxner-Höck et al. 2013:511, Plate 22.1, fig. 5).

**Locality, Stratigraphy:** The Tagay-1 section (layers 9, 7) and test sample Kossler, from Olkhon Island, Baikal Region, Siberia; Tagay Formation; Early/Middle Miocene transition.

**Diagnosis:** The small ground squirrel *Spermophilinus debruijni* nov. spec. has a very short P4 by missing the anteroloph and parastyle. The upper molars M1/2 have a sub square shape and a concave occlusal surface. The three distinct cones are higher than the four lophs. The oblique metaloph is constricted before reaching the protocone. Lower teeth have a rhomboidal outline, specifically p4 and m1. The posterolingual corner of m2/3 is strongly flattened by incorporation of the entoconid into the posterolophid. Metalophid of m2/3 is short, thus the basins of trigonid and talonid are fused. The main characteristics of *Spermophilinus debruijni* nov. spec. are: very low tooth-crowns, the lack of a distinct hypocone and entoconid, and of any distinct conules/conulids (i.e. anteroconule, protoconule, metaconule, anteroconid, mesoconid and mesostylid). Upper P4 and M1–M3 have three roots, lower p4 has two roots, and m1–3 have three roots.

**Differential diagnosis:** Tooth morphology of *Spermophilinus debruijni* nov. spec. differs from all *Spermophilinus*-species by the absence of: hypocone, entoconid and any marked conules/conulids, such as anteroconule, protoconule, metaconule, anteroconid, mesoconid and mesostylid, and by the extremely short P4 (parastyle absent). The molar measurements are within the size range of *S. besana* Cuenca Bescós, 1988 but smaller than teeth of *S. bredai* (Von Meyer, 1848), *S. turolensis* de Bruijn and Mein, 1968, *S. giganteus* de Bruijn, Dawson and Mein, 1970 and *S. mongolicus* Qiu and Li, 2016.

*S. besana* of similar size with *S. debruijni* nov. spec. differs by a more pronounced groove (Spanish = *besana*) anterior to the protoconid of lower molars and by more marked conules/conulids.

**Description of the holotype**: (Fig. 7c) It is a right M1/2 (ZIN 106455) with subsquare outline and concave occlusal surface. The three main cones (paracone, metacone, protocone) are higher than the four lophs (anteroloph, protoloph, metaloph and posteroloph). The hypocone is incorporated into the protocone. The drop-shaped labial cones are continuous with the protoloph and metaloph, respectively. The anteroloph attaches the antero-basal part of the protocone. The transverse protoloph attaches the protocone at its anterior part. The forward directed metaloph is constricted before reaching the middle part of the protocone. The transverse posteroloph lingually turns forward and attaches the protocone at its posterior base. Both conules, the protoconule and metaconule are absent. Three synclines are separated by the protoloph and the incomplete metaloph, respectively. The 1^st^ and 3^rd^ synclines are narrower than the 2^nd^ one. The root fragments suggest a three-rooted tooth with one lingual and two labial roots.

Description of the paratypes and additional material

**P4** (Fig. 7a) is short, it has no anteroloph and no parastyle. The labial cones, paracone and metacone, are accentuated, the lingual protocone is largest of all cones. The hypocone is incorporated into the protocone. The transverse protoloph attaches the anterior part of the protocone, the metaloph is slightly directed forward and contacts the protocone at its basal middle part. Both, protoconule and metaconule are absent. The posteroloph extends from the base of the metacone toward lingual, turns forward at the postero-lingual edge and attaches the posterior part of the protocone. P4 has three roots.

**M1/2** (Fig. 7b): The dental pattern is almost identical with the holotype. One M1/2 is damaged at its postero-labial edge. There are three roots.

**M3** (Fig. 7d) has a triangular occlusal outline with rounded corners. There are only two distinct cones (paracone and protocone) and two transverse lophs (anteroloph and protoloph). The anteroloph attaches the antero-basal part of the protocone. The wide central basin is enclosed by the protoloph and by the posteroloph, in which the protocone and metacone are incorporated. M3 has three roots.

**p4** (Fig. 7e): The two available p4 show similar dental characteristics and size. There are three conids (metaconid, protoconid and hypoconid) of almost equal elevation. Metaconid and protoconid are separated by a deep notch. The low and thin longitudinal ectolophid connects protoconid and hypoconid. The posterolophid is strong and almost as high as the conids; it derives from the hypoconid, encircles the talonid basin along the posterior and lingual margin of the tooth, where it gradually becomes lower and finally attaches to the base of the metaconid. The entoconid is incorporated into this marginal ridge. The p4 has no anterolophid, anteroconid, mesoconid, entoconid, mesostylid and no trigonid basin. The small labial sinusid is lingually bound by the ectolophid. Two roots are present, one anterior and one posterior.

**m1** (Fig. 7f): The occlusal outline is rhomboidal. The m1 is strongly damaged in its anterior and labial part. The tooth fragment suggests the following structures: three conids (metaconid, protoconid and hypoconid), the ectolophid and the prominent posterolophid with incorporated entoconid. Both, protoconid and metaconid are connected by two short ridges, the anterolophid and the metalophid, which enclose the small trigonid basin. The ectolophid is very short. The sinusid-region is damaged. The posterolophid of m1 resembles that of p4. Anteroconid, mesoconid, entoconid and mesostylid are absent. Roots are not preserved.

**m2/3** (Fig. 7g): Two available specimens are of similar shape, one (ZIN 106463) is strongly worn. The occlusal outline is almost triangular, with an obtuse-angled protoconid region, and a long posterolophid ranging along the postero-lingual margin from the hypoconid to the metaconid. The metaconid is the highest conid. The anterolophid ranges from the top of the metaconid in slight backward direction toward the protoconid, but does not contact the latter. The unworn tooth (ZIN 106462; Fig. 7g) shows a groove separating the lingual end of the anterolophid and the protoconid. This groove disappears with increasing wear. The metalophid is short; it incompletely divides the trigonid basin from the taloned basins. The protoconid and hypoconid are connected by the longitudinal ectolophid. The sinusid is very shallow. The m2/3 has no distinct mesoconid, entoconid and no mesostylid. There are three roots, two in anterior position, one in posterior position.

**Discussion:** The ground squirrel from the Tagay locality shares the basic tooth pattern with the genus *Spermophilinus.* The genus comprises five morphologically similar species, which differ primarily in size. Ordered from small to large these species are: *S. besana, S. bredai, S. mongolicus, S. turolensis* and *S. giganteus. Spermophilinus* species were distributed all over Europe from the Early Miocene to the Pliocene. The stratigraphic ranges are: *S. besana* (Orleanian, MN3–MN5, Early Miocene), *S. bredai* (Astaracian–Turolian, MN6–MN11, Middle to Late Miocene), *S. turolensis* (Turolian, MN12–MN13, Late Miocene) and *S. giganteus* (Ruscinian, MN14, Pliocene). Findings from Anatolia (Turkey) demonstrate the oldest (Astaracian, MN 2, Early Miocene) and youngest (Turolian, MN9, Late Miocene) occurrences of *S. besana*, respectively, and also *S. bredai* and *S. turolensis* are reported from Turkey (Bosma et al. 2013, 2019). Hitherto, only one species, *S. mongolicus* (Middle to Late Miocene), was described from China. It resembles *S. bredai* in size (Qiu and Li 2016).

*Spermophilinus* from Tagay is a small species, comparable in tooth sizes with *S. besana,* which is the smallest and oldest *Spermophilinus* species known from Europe and Turkey. The significant differences in tooth morphology of the Siberian specimens justifies differentiation from all known species. Therefore it is described as *S. debruijni* nov. spec*.* Small size and relatively primitive tooth pattern suggest an Early Miocene rather than a Middle Miocene age.

Sciuridae indet.

(Fig. 8)

**Fig. 8:**
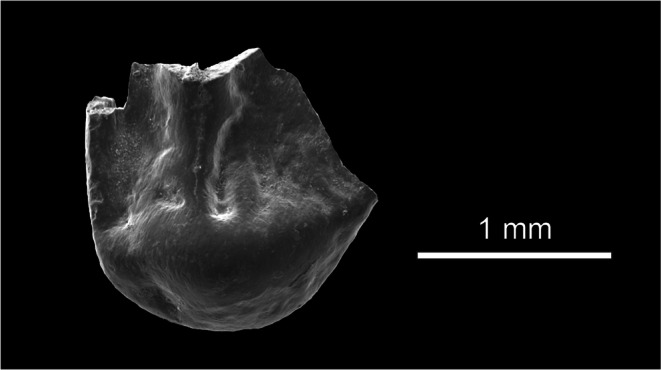
Sciuridae indet. of the Tagy-1 section (layer 9; Olkhon Island, Baikal Region, Siberia).

**Locality, Stratigraphy:** The Tagay-1 section (layer 9) from Olkhon Island, Baikal Region, Siberia; Tagay Formation; Early/Middle Miocene transition.

**Material:** One left fragmentary M3 (ZIN 106464) from bed 9 of the Tagay-1 section (collected by Erbajeva and colleagues in 2014).

**Description and remarks:** The molar fragment is the lingual part of a left M3 (Fig. 8), with a prominent protocone, parts of the anteroloph, protoloph and posteroloph. Close to the posteroloph the central basin shows wrinkled struktures. The strongly damaged tooth suggests larger size and more structured dental pattern than M3 of *Spermophilinus debruijni* nov. spec., and therefore is described as Sciuridae indet.

## Conclusions

Olkhon Island of Lake Baikal stands out for its unique nature and origin. The soft wavy relief was formed by tectonic and geomorphologic processes. The original relief of the ancient peneplain, built up by Paleozoic rocks, was leveled during the Cretaceous and Paleogene, leaving small basins, which were filled up by Neogene sediments. During Quaternary tectonic processes, Olkon Island began to separate from the hinterland and to sink, dipping toward west, where parts of the island subsided into Lake Baikal. The coastal zone was exposed to coastal erosion, forming cliffs of the basement rocks, separated by wide open bays, where Neogene sediment sequences became exposed. In one of these bays, Tagay Bay, the Tagay-1 section is located. From bottom to top of the section 17 layers were identified and studied. They differ in lithology, sediment structures, geochemistry and in the fossil content.

This paper focuses on the rodent families Aplodontidae, Mylagaulidae and Sciuridae. The respective fossils were recovered from two individual layers of the Tagay-1 section, layers 9 and 7.

The species *Ansomys* sp. (Aplodontidae) is represented by three teeth (from layers 9 and 7), *Lamugaulus olkhonensis* Tesakov and Lopatin, 2015 (Mylagaulidae) by one tooth (from layer 9), Sciuridae indet. by one tooth (from layer 9) and *Spermophilinus debruijni* nov. spec. (Sciuridae) by nine teeth (from layers 9 and 7) and 1 tooth from sample Kossler (which is correlative with layers 9–7 of section Tagay-1from 2014).

The few specimens of *Ansomys* sp. and *L. olkhonensis* mirror the poor record of Aplodontidae and Mylagaulidae in the Neogene of Eurasia. In Asia, *Ansomys* ranges from the Oligocene to the Late Miocene, and is best known from Early and Middle Miocene assemblages of Nei Mongol (China). *Ansomys* sp. from the Tagay-1 section is similar to *A. orientalis* from the Early Miocene of China but seems more advanced. *L. olkhonensis* is only known from the Tagay locality, it belongs to the rodent family Mylagaulidae, which migrated from North America to Asia in the course of the Miocene, and suggests an Early Miocene age (Tesakov and Lopatin 2015). The well-represented Sciuridae species *S. debruijni* nov. spec. is comparable in tooth size with *Spermophilinus besana*, the smallest and oldest *Spermophilinus* species known from Europe and Asia Minor, but tooth characteristics differ considerably. However, small size and relatively primitive tooth pattern suggest an Early- rather than a Middle Miocene age. The final biostratigraphic placing of the Tagay fauna will be possible after completing the work on all small mammal fossils.

## Data Availability

All data generated or analysed during this study are included in this published article.
